# The novel subclusters based on cancer-associated fibroblast for pancreatic adenocarcinoma

**DOI:** 10.3389/fonc.2022.1045477

**Published:** 2022-12-05

**Authors:** Guojie Zhao, Changjing Wang, Jian Jiao, Wei Zhang, Hongwei Yang

**Affiliations:** ^1^ The Seventh Department of General Surgery, HanDan Central Hospital, Handan, Hebei, China; ^2^ The Department of Gastrointestinal surgery, The Third Hospital of Hebei Medical University, Shijiazhuang, Hebei, China; ^3^ The First Department of Oncology, HanDan Central Hospital, Handan, Hebei, China

**Keywords:** pancreatic adenocarcinoma, immune features, machining learning, prognosis, immunotherapy, subclusters

## Abstract

**Introduction:**

Pancreatic adenocarcinoma (PAAD) is a fatal disease characterized by promoting connective tissue proliferation in the stroma. Activated cancer-associated fibroblasts (CAFs) play a key role in fibrogenesis in PAAD. CAF-based tumor typing of PAAD has not been explored.

**Methods:**

We extracted single-cell sequence transcriptomic data from GSE154778 and CRA001160 datasets from Gene Expression Omnibus or Tumor Immune Single-cell Hub to collect CAFs in PAAD. On the basis of Seurat packages and new algorithms in machine learning, CAF-related subtypes and their top genes for PAAD were analyzed and visualized. We used CellChat package to perform cell–cell communication analysis. In addition, we carried out functional enrichment analysis based on clusterProfiler package. Finally, we explored the prognostic and immunotherapeutic value of these CAF-related subtypes for PAAD.

**Results:**

CAFs were divided into five new subclusters (CAF-C0, CAF-C1, CAF-C2, CAF-C3, and CAF-C4) based on their marker genes. The five CAF subclusters exhibited distinct signaling patterns, immune status, metabolism features, and enrichment pathways and validated in the pan-cancer datasets. In addition, we found that both CAF-C2 and CAF-C4 subgroups were negatively correlated with prognosis. With their top genes of each subclusters, the sub-CAF2 had significantly relations to immunotherapy response in the patients with pan-cancer and immunotherapy.

**Discussion:**

We explored the heterogeneity of five subclusters based on CAF in signaling patterns, immune status, metabolism features, enrichment pathways, and prognosis for PAAD.

## Introduction

Pancreatic adenocarcinoma (PAAD) is a serious threat to people’s life and health due to its high degree of malignancy and poor prognosis. According to the latest epidemiological data, pancreatic cancer is the 12th most common tumor in the world but the fourth most deadly cancer worldwide (1, 2). Pancreatic ductal adenocarcinoma (PDAC) is the most common histologic type of PAAD. PDAC has low resection rate, insensitive radio chemotherapy, and poor prognosis, and the 5-year survival rate is less than 7% (1, 3). PAAD develops gradually from genetic abnormality to abnormal cell proliferation and precancerous lesions and then to minimal early carcinoma, which takes a very long time, about 5–20 years. However, it only takes 6 to 20 months to develop from a small tumor to a significant mass and then to the advanced stage. In addition, because of the painless and insidious growth of pancreatic masses, most patients with pancreatic cancer are already diagnosed in advanced stages. Therefore, the study of the pathogenesis and progression of PAAD and the search for suitable bimolecular targets are of great significance to enrich the treatment strategies of pancreatic cancer and improve the prognosis of patients.

The occurrence and development of tumors are closely related to their living environment, and the internal environment composed of tumor cells, mesenchymal cells, immune cells, vascular endothelial cells, and extracellular matrix (ECM) is called tumor microenvironment (TME) (4). During the development of PAAD, a microenvironment is formed, which is favorable for the survival, proliferation, and distant metastasis of PAAD cells (5). The poor prognosis of pancreatic cancer may be associated with specific biological characteristics, such as significant interstitial fibrosis (6). In recent years, researchers have paid more and more attention to the stroma of PAAD (7, 8). Dense fibrous tissue surrounding tumor cells is an important histologic feature of PDAC (9–11). The main components of interstitium include ECM, immune cells, endothelial cells, and cancer-associated fibroblast (CAF) (12), and stroma microenvironment cells interact with tumor cells in a complex way (13). TME can determine the biological behavior of the tumor, which, in turn, affects patient prognosis. Therefore, understanding the biological characteristics of TME is crucial for understanding the biological behavior of PAAD (14).

Tumor stroma cells are complex, and interstitial cells interact with each other (7, 15). The relatively abundant cell components in the stroma are CAFs, and CAFs are closely related to the significant proliferation of connective tissue of PAAD cells. CAFs are considered to be fibroblasts that produce ECM, cytokines, chemokines, and growth factors, with the primary function of promoting tumor progression (16). However, some targeted therapy studies on CAFs suggested that removal of CAFs can promote tumor progression or metastasis (17, 18), suggesting significant heterogeneity of CAFs within tumors (19), that is, some CAF subgroups may play a role in inhibiting tumor progression. A large number of single-cell transcriptome sequencing studies have further clarified the significant heterogeneity of CAFs within and between tumors, as well as the functional classification of CAFs (20, 21). Currently, commonly accepted cancer-associated fibroblast (CAF) are categorized as myofibroblastic CAFs (myoCAFs) and inflammatory CAFs (iCAFs). myoCAFs are mainly distributed around tumor cells and are mainly related to the generation of ECM. Some reports suggested that some subgroups of myoCAFs may be involved in immune regulation (22). iCAFs mainly secrete cytokines and chemokines to act on tumor cells. In addition, other small CAF subsets, such as apCAF (20) and LRRC15 (+) myoCAFs (21), were identified. Of course, different subsets of cells perform different functions, and as single-cell sequencing technology continues to mature, more functional subsets of CAFs may be discovered. At present, single-cell sequencing studies suggested that representative markers of myoCAFs were Alpha-smooth muscle actin (α-SMA), periostin, and matrix metallopeptidase-11 (MMP-11); representative markers of iCAFs were Interleukin-6 (IL-6), C-X-C Motif Chemokine Ligand 12 (CXCL12) stromal cell-derived factor-1 (SDF-1), and Platelet-derived growth factor receptors-beta (PDGFR-β); and fibroblast activation protein-alpha (FAP-α) was a co-expression marker of two types of CAFs (20). However, the origin, function, and biological characteristics of CAFs need to be further studied.

In the current study, we gained single-cell sequence transcriptomic data from public databases. We carried out comprehensive analysis to generate five CAF subclusters and explore the differences among them. This will provide new insights into the treatment of PAAD.

## Materials and methods

### Study design and data collection

**Figure 1:**
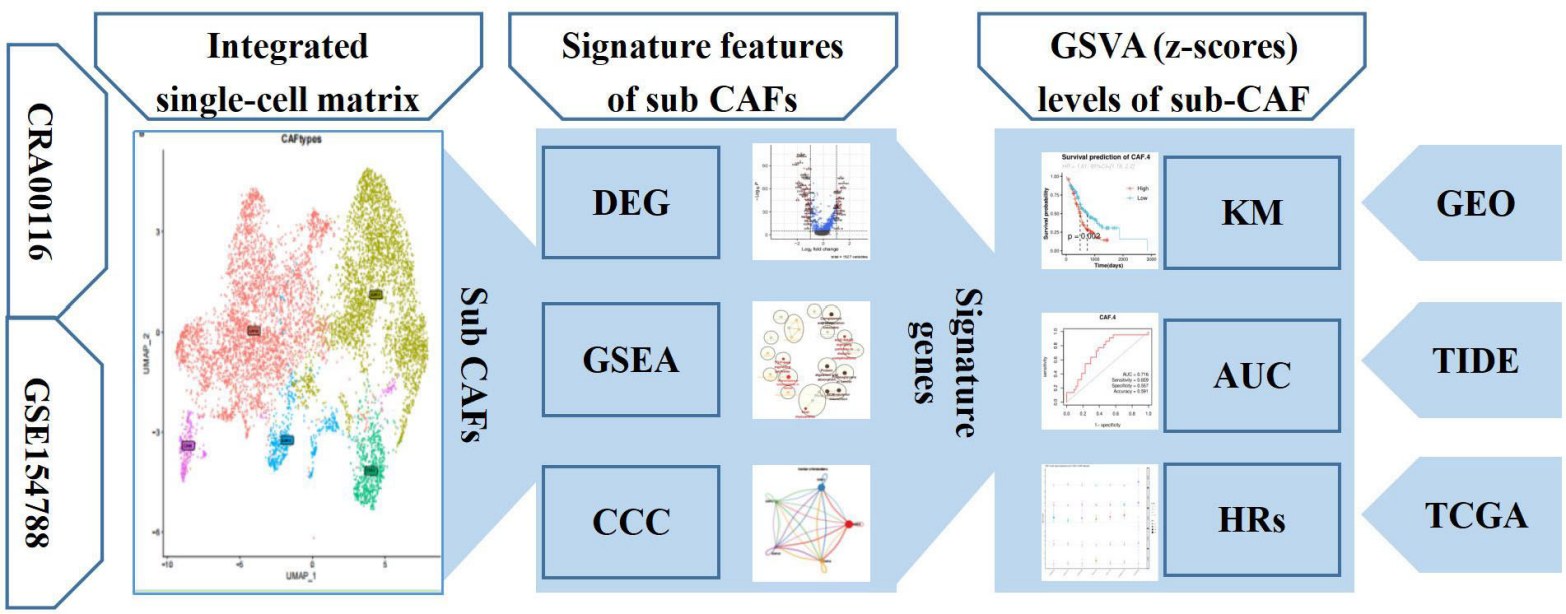
The workflow diagram depicting collection of data and processing of the analysis to show the framework of our study.

The flowchart of present study is shown in **Figure 1**. Single-cell sequence transcriptomic data from the GSE154778 and CRA001160 datasets were collected to analyze the fibroblast cells (23, 24). Full details can be downloaded from Gene Expression Omnibus (GEO) (www.ncbi.nlm.nih.gov/geo) and Tumor Immune Single-cell Hub (TISCH; http://tisch.comp-genomics.org/) databases (25). Among them, we extracted CAF cells to analyze the features. In addition, seven bulk-sequence data for PDAC—TCGA (n = 146), ICGC-PACA-AU (n = 267), GSE71729 (n = 125), GSE62452 (n = 66), GSE57495 (n = 63), ICGC-PACA-CA (n = 182), and E_MTAB_6134 (n = 50)—were enrolled from GEO and The Cancer Genome Atlas (TCGA) databases based on previous data (26). Pan-cancer dataset with 31 cancer types was also collected to verify the features of the single-cell subsets. All data generated or analyzed during this study are freely available in the previous publications. Last, to get the immune features of the subset of single-cells, 10 cohorts with different tumors before or after immunotherapy [immune checkpoint blockade (ICB)] were collected in Tumor Immune Dysfunction and Exclusion (TIDE) database to further analysis (27).

### Subset for fibroblast cells

The Seurat R package was used to visualize the CAF cells from two cohorts (28), including the 1,656 CAFs in GSE154778 and 6,228 CAFs in CRA001160. Top 2,000 genes were integrated by the method of canonical correlation analysis (CCA) to integrate CAFs for dimensionality reduction cluster analysis (Resolution = 0.1, N = 5) (29). We also performed ScaleData, RunPCA, DimPlot, and t-SNE (t-distributed stochastic neighbor embedding) based on R packages to analyze and visualize results.

### Cell–cell communication analysis

The CellChat R package with full of ligand–receptor interactions can analyzed the intercellular communication networks between different cell clusters in the single-cell dataset (30). To access the major signaling inputs and outputs among subsets and other TME cells, the CellChatDB.human,netVisual_circle and netVisual_bubble functions were used to show the strength or weakness of cell–cell communication networks from the CAF subclusters to other different cell clusters in single-cell dataset.

### Identification of the marker genes of CAF cell subtypes

FindAllMarkers function was used to list the markers of subclusters of CAF (31). The min.pct and logfc.threshold functions were all set as 0.25. The AddModuleScore function could obtain the signature scores based on differentially expressed genes (DEGs) (32). The dot plot function was performed to show the top highest gene expressions in subcluster (33). The FeaturePlot function was used to show the distribution of specific signatures of subcluster scores. The volcano plot based on the marker genes among different subsets of CAF was displayed.

### Functional enrichment analysis for CAF subsets

The significant Kyoto Encyclopedia of Genes and Genomes (KEGG) pathways and Gene Ontology (GO) functions were detected by the clusterProfiler R package (34) based on marker genes among different subsets of CAF. To cluster the special pathways, the Cytoscape enrichment map function was performed in the Cytoscape software (35).

### Prognosis analysis and prediction analysis of subsets

We first performed the gene set variation analysis (GSVA) (36) based on the subset signatures of CAF subsets to get the enrichment scores for these subclusters of CAF in the PDAC bulk sequence. On the basis of their prognostic information, we analyzed the prognosis features of subsets of CAF enrichment score in the cohorts from TCGA and GEO. The cutoff values of different NMF cell signatures in the different public datasets were determined by the survminer R package (37) used to plot Kaplan–Meier (K-M) curves. The prediction value of subsets of CAF for immunotherapy also was performed by the receiver operating characteristic (ROC) analysis. The ComplexHeatmap (38) or pheatmap (39) packages in R visualize the pooled values of CAF in these cohorts.

### Gene expression detecting using quantitative real-time PCR assays

The human pancreatic CAF-stellate cell named CAF118 was supplied by Neuromics (Edina, USA) and was cultured using Stem Cell Complete Low Serum Media (Neuromics, Edina, USA). The human pancreatic cell HPC-Y5 was purchased from National Collection of Authenticated Cell Cultures and was cultured in 90% MEM Eagles with Earle’s Balanced Salts (EME-EBSS) with 10% FBS (fetal bovine serum). The human pancreatic cancer cell line SW1990 was purchased from Procell (Wuhan, China) and cultured in 90% LEIBOVITZ (L-15) with 10% FBS. After extracting the total RNA of the cell lines by the RNAsimple Total RNA Kit (Tiangen, China), we reverse-transcribed RNA to acquire cDNA using the PrimeScript RT reagent Kit (Takara, Otsu, Japan). Finally, on the basis of the premixed system of 2 μl of cDNA with SYBR Premix Ex Taq (Takara, Otsu, Japan) and primers, we detected the expression values of related genes in cell lines by an Applied Biosystems StepOne Plus Real-Time PCR system (Life Technologies, Grand Island, NY, USA). The primers of the target gene were supplied by Sangon Biotech (Shanghai, China). The sequences of the primers used are listed in **Table 1**.

**Table 1:** The primer sequences in PCR analysis.

Symbol	Sequences (5′-3′)
ADM-F	CTGATGTACCTGGGTTCGCT
ADM-R	ATGTCCTGGGGCCGAATAAG
Eno2-F	CTCTGTGGTGGAGCAAGAGA
Eno2-R	ATTGATCACGTTGAAGGCCG
ERO1A-F	TTGGATCTGCTGGTGGTCAT
ERO1A-R	TCCCTTGACCAGAAGCCAAA
BNIP3-F	CGCAGACACCACAAGATACC
BNIP3-R	GCGCTTCGGGTGTTTAAAGA
UPP1-F	TTGACTGCCCAGGTAGAGAC
UPP1-R	TGCCTGCTCTGTTATGACCA
Actin-F	ACTTCGAGCAAGAGATGGCC
Actin-R	GCTGATCCACATCTGCTGGA

### Statistical analysis

Routine statistical analyses of the present study were performed in R 4.0 software. The relationships of sub-CAF with other special genes were calculated by the Spearman’s rank correlation. The K-M method, log-rank test, and Cox regression analysis were performed to detect the prognosis of subset of CAF in the OS (overall survival) and RFS (relapse-free survival) in patients with Pancreatic ductal adenocarcinoma (PDAC) and other tumor. The area under the ROC curve was used to estimate the diagnostic value of GSVA score of subset of CAF. A two-sided p-value below 0.05 was considered statistically significant.

## Results

### Identification of five CAF-related subtypes for PDAC

**Figure 2:**
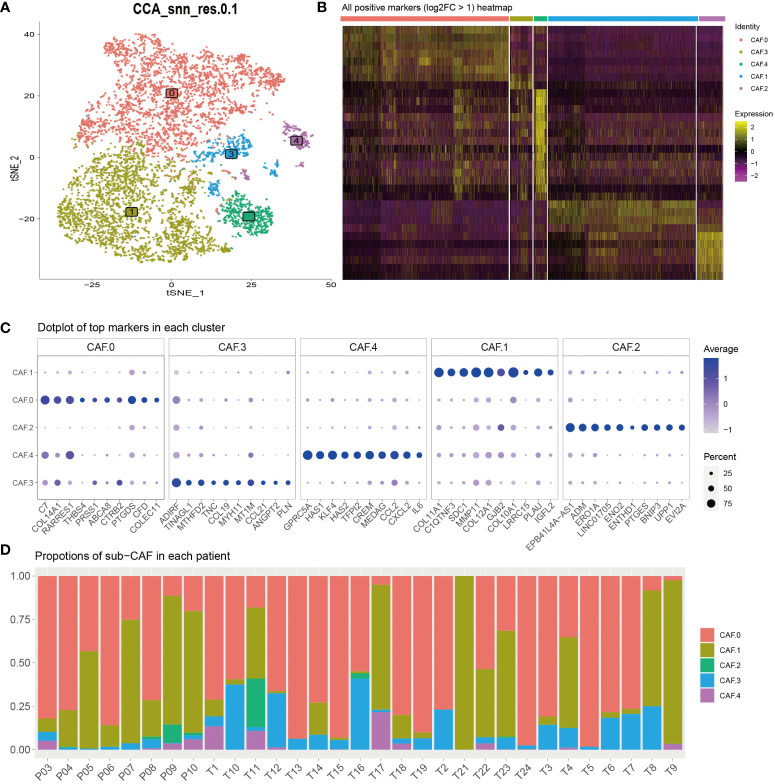
**(A)** To characterize the CAF subpopulations in PDAC, we performed unsupervised clustering analysis and showed that CAFs, which were categorized into five subclusters (C0, C1, C2, C3, and C4). **(B)** All-positive markers (log2FC > 1) heatmap of CAF cell subtypes. The colors of the top bar represent the different subclusters. Yellow indicates higher expression, and purple indicates lower expression. **(C)** Dot plot of top 10 markers in each cluster. The color represents the average expression. The size of the circle represents the percent. **(D)** Proportions of sub-CAF in each patient. The axis represents the ratio of different subclusters for each patient. The colors of the bar represent the different subclusters.

Recent SCNA-SEQ studies of human PDAC have shown that intra-tumor heterogeneity of PDAC is key to the analysis of tumor-related mechanisms. Extensive fibrous proliferation caused by CAFs is common in PDAC. In clinical practice, we often encounter PDAC tumors with unique histological characteristics. To characterize the CAF subpopulations in PDAC, we performed unsupervised clustering analysis (**Figure 2A**). The all-positive expressed markers (log2FC > 1) are shown in **Figure 2B** and **Supplementary Table S1**. The results showed that CAFs were divided into five subclusters based on their marker genes (**Figure 2C**): CAF-C0 (by marker genes C7 and PTGDS), CAF-C1 (by marker genes COL11A1 and COL10A1), and CAF-C2 (by marker genes EPB41L4A-AS1 and ENO2). Proportions of sub-CAF in each patient is different, and that verifies the features for the single-cell subsets (**Figure 2D**).

### CAF subclusters exhibited distinct signaling patterns

**Figure 3:**
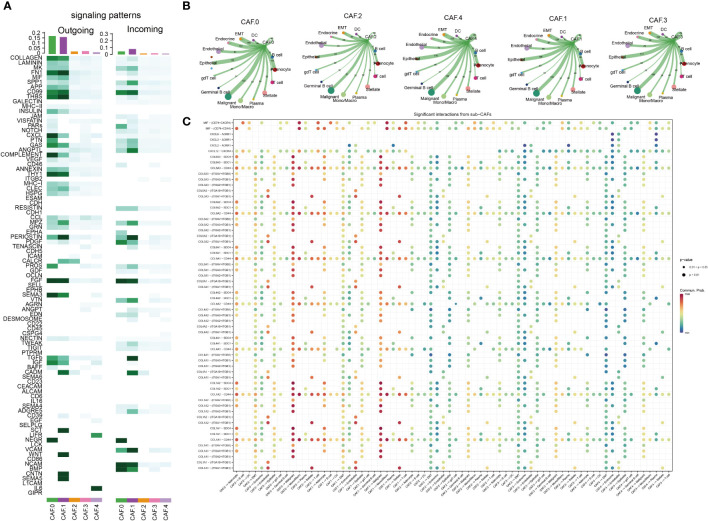
Cell–cell communication analysis. **(A)** The major signaling inputs and outputs among subsets. **(B)** Detailed view of the ligand–receptor expressed by each cell type and the other cell types for each CAF subclusters. The thicker the lines, the greater the number/intensity of ligand receptor. The size of the dots represents the number of cells in the subpopulation. **(C)** Bubble plot showing the ligand–receptor interactions between CAF clusters and cells. P-values are indicated by circle size. Communication proportion is indicated by color. The redder the color, the more important the interaction.

The major signaling inputs and outputs among subclusters were different. The characteristics of signaling patterns within each CAF subgroup were different. The results showed that subcluster CAF-C0 was related to CD99, MK, PDGF, NEGR, NCAM, BMP, and CD46; CAF-C1 was related to FN1, CD99, MK, PDGF, NEGR, NCAM, BMP, and CD; CAF-C2 was related to TGB2; whereas CAF-C4 was related to ITGB2 (**Figure 3A**). The cross-linking between CAF and 14 kinds of main TME cells in each subcluster was also different (**Figures 3B, C**). CAF-C0 was closely related to adenocyte, epithelial-to-mesenchymal transition (EMT), endocrine, epithelial, and malignant, whereas CAF-C1 was closely related to adenocyte, EMT, endocrine, epithelial, and malignant. CAF-C2 was closely related to adenocyte, EMT, endocrine, epithelial, and malignant, whereas CAF-C3 was closely related to adenocyte, EMT, endocrine, epithelial, and malignant. CAF-C4 was associated with adenocyte, EMT, endocrine, epithelial, malignant, endothelial, and plasma.

### CAF subclusters exhibited distinct immune and metabolism features

**Figure 4:**
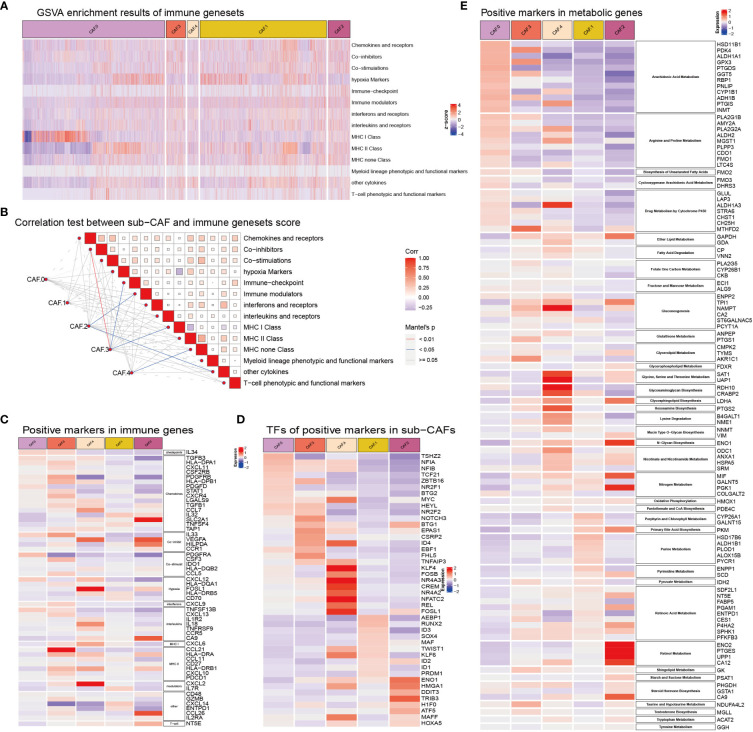
**(A)** GSVA enrichment results of immune gene sets in sub-CAFs. The z-score represents the rating. **(B)** Correlation test between sub-CAF and immune gene sets score. The color of the lines indicates the Mantel’s p-value. The color of the box represents the correlation. **(C)** Positive markers in immune genes in sub-CAFs. The redder the color, the higher the expression of the gene in the CAF cluster. The bluer the color, the lower the expression of the gene in the CAF cluster. **(D)** TFs of positive markers in sub-CAFs. The bluer the color, the lower the expression of the gene in the CAF cluster. **(E)** Positive markers in metabolic genes in sub-CAFs. The bluer the color, the lower the expression of the gene in the CAF cluster.

Some subsets based on CAFs were significantly correlated with immune gene sets, such as CAF-C2 and immune modulators, other cytokines, C3 and co-inhibitors, immune checkpoint, MHC class I, and C4 and MHC non-class (**Figures 4A–D**). The expression of metabolism-related genes of CAF in each subclusters was different (**Figure 4E**). The genes related to arachidonic acid metabolism (HSD11B1, PDK4, ALDH1A1, GPX3, PTGDS, GGT5, RBP1, PNLIP, CYP1B1, ADH1B, PTGIS, and INMT), arginine and proline metabolism (PLA2G1B, AMY2A, PLA2G2A, ALDH2, MGST1, PLPP3, CDO1, FMO1, and LTC4S), Cyclooxygenase arachidonic acid metabolism (FMO3 and DHRS3), and drug metabolism by cytochrome P450 (GLUL, LAP3, ALDH1A3, STRA6, CHST1, and CH25H) were highly expressed in CAF-C0. The genes related to purine metabolism (HSD17B6, ALDH1B1, PLOD1, ALOX15B, and PYCR1) and pyrimidine metabolism (ENPP1 and SCD) were highly expressed in CAF-C1. Genes associated with sugar synthesis and metabolism, such as N-glycan biosynthesis (ENO1), oxidative phosphorylation (HMOX1), primary bile acid biosynthesis (PKM), retinol metabolism (ENO2, PTGES, UPP1, and CA12), starch and sucrose metabolism (PSAT1), and steroid hormone biosynthesis (PHGDH, GSTA1, and CA9), were highly expressed in CAF-C2. The genes related to glycerolipid metabolism (CMPK2, TYMS, and AKR1C1), sphingolipid metabolism (GK), taurine and hypotaurine metabolism (NDUFA4L2), and testosterone biosynthesis (MGLL) were highly expressed in CAF-C3. The metabolic genes related to lipid and amino acid [such as ether lipid metabolism (GAPDH and GDA); fatty acid degradation (CP and VNN2); gluconeogenesis (ENPP2, TPI1, NAMPT, CA2, and ST6GALNAC5); glycine, serine, and threonine metabolism (SAT1 and UAP1); glycosaminoglycan biosynthesis (RDH10 and CRABP2); hexosamine biosynthesis (PTGS2), lysine degradation (B4GALT1 and NME1); and nicotinate and nicotinamide metabolism (ODC1, ANXA1, HSPA5, and SRM)] were highly expressed in CAF-C4.

### CAF subclusters exhibited distinct enrichment pathways

**Figure 5:**
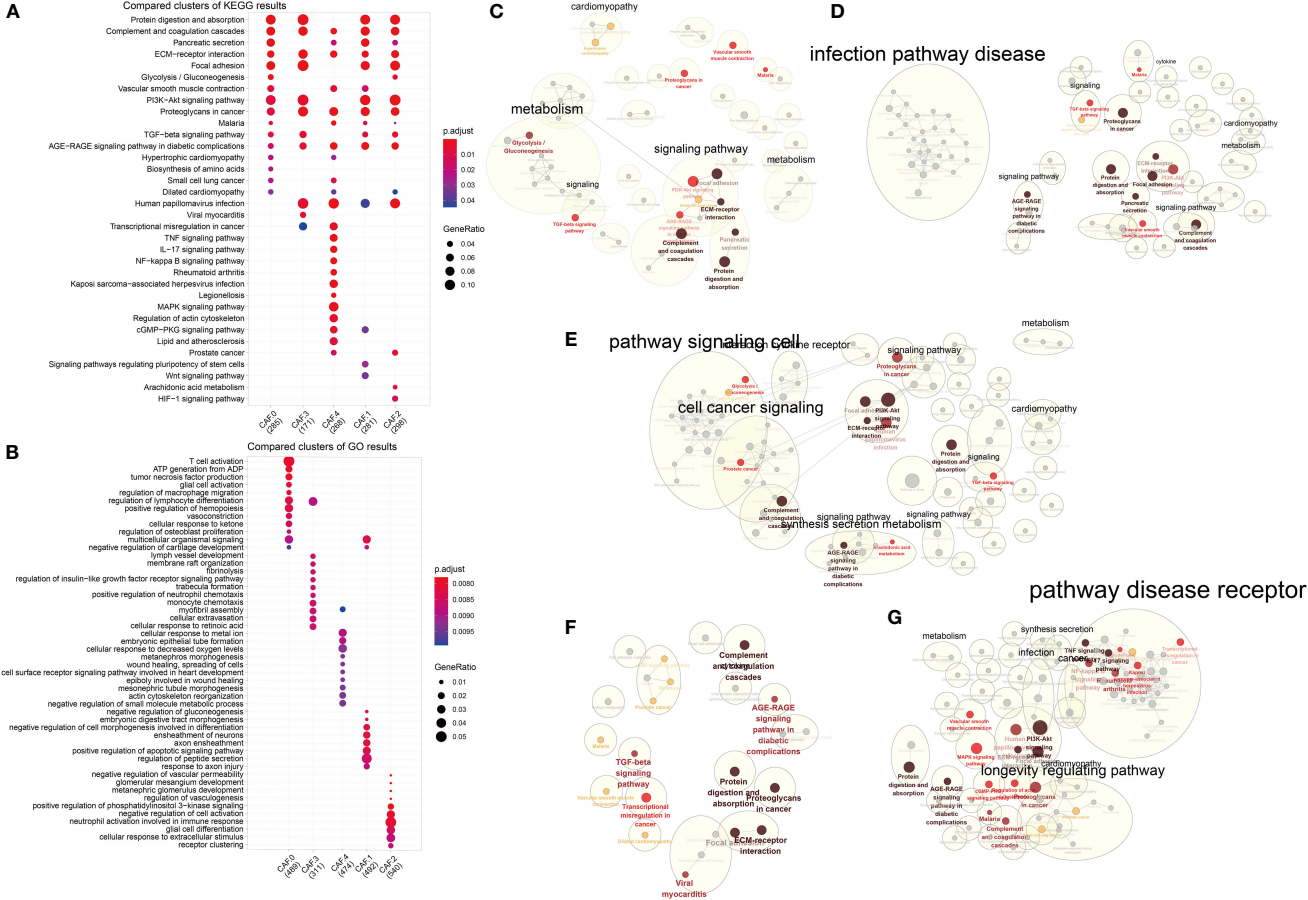
Functional enrichment analysis for CAF subsets. **(A)** Compared clusters of KEGG results. The color represents the P-value, and the size of the circle represents the ratio of genes. **(B)** Compared clusters of GO results. The color represents the P-value, and the size of the circle represents the ratio of genes. **(C–G)** Networks of functional enrichment analysis elaborated by Cytoscape.

GO and KEGG analysis suggested differences in their biological functions of the five subclusters (**Figures 5A, B**) and **Supplementary Table S2**. Interestingly, all five subtypes were enriched in four pathways: complement and coagulation cascades, ECM–receptor interaction, proteoglycans in cancer, and AGE-RAGE signaling pathway in diabetic complications (**Figure 5A**). As for CAF-0, there were highly expressed genes involved in T cell activation, ATP generation from ADP, tumor necrosis factor production, vasoconstriction, cellular response to ketone, biosynthesis of amino acids, and so on. For CAF-C1, there were highly expressed genes involved in regulation of peptide secretion, positive regulation of apoptotic signaling pathway, negative regulation of cell morphogenesis involved in differentiation, Wnt signaling pathway, and signaling pathways regulating pluripotency of stem cells. For CAF-C2, there were highly expressed genes involved in cellular response to extracellular stimulus, neutrophil activation involved in immune response, negative regulation of cell activation, HIF-1 signaling pathway, and arachidonic acid metabolism. For CAF-C3, there were highly expressed genes involved in monocyte chemotaxis and regulation of insulin-like growth factor receptor signaling pathway. For CAF-C4, there were highly expressed genes involved in cellular response to decreased oxygen levels, cellular response to metal ion, negative regulation of small molecule metabolic process, mitogen-activated protein kinase (MAPK) signaling pathway, tumor necrosis factor (TNF) signaling pathway, and IL-17 signaling pathway. We established networks to elaborate how related genes were functionally enriched (**Figures 5C–G**).

### Survival analysis of different CAF subclusters

**Figure 6:**
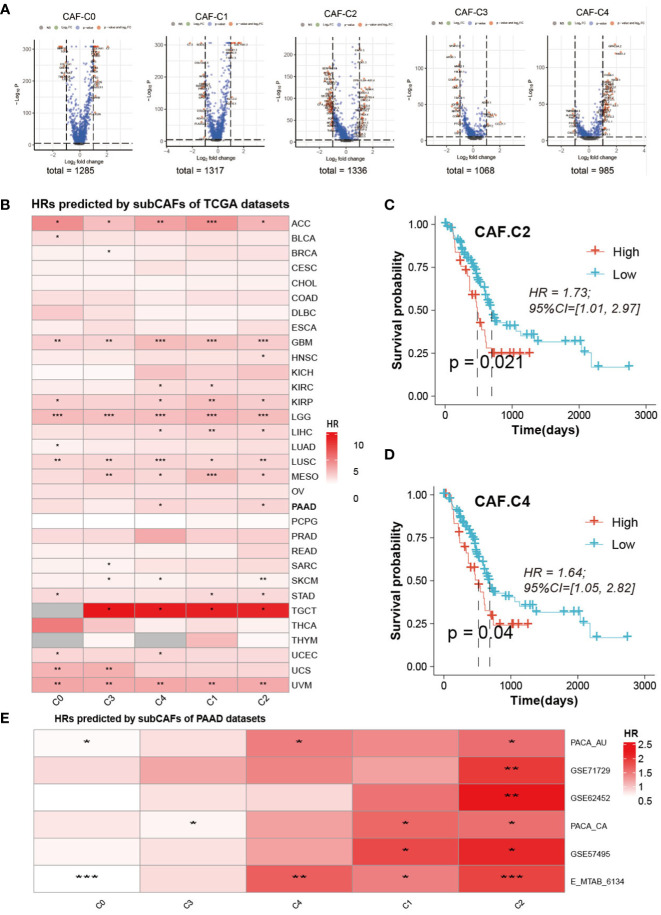
Prognosis analysis and Prediction analysis of subsets. **(A)** The volcano figure of differential expression analysis of five CAF-clusters. **(B)** HRs predicted by subCAFs of TCCA datasets. The color represents the HR value. *P < 0.05, **P < 0.01, ***P < 0.001. **(C)** Survival curve predicted for CAF-C2. **(D)** Survival curve predicted for CAF-C4. **(E)** HRs predicted by subCAFs of PAAD datasets. The color represents the HR value. *P < 0.05; **P < 0.01; ***P < 0.001.

Volcanic maps of differential genes for comparison of CAF between two groups were shown in **Figure 6A**. We can see the distribution of hazard ratios (HRs) based on sub-CAFs for tumors in TCGA database from **Figure 6B**. For ACC (adrenocortical carcinoma), GBM (glioblastoma multiforme), LGG (brain lower-grade glioma), LUSC (lung squamous cell carcinoma), and UVM (uveal melanoma), HRs predicted by sub-CAFs were all statistically significant. We collected PAAD data from seven databases and analyzed the correlation between CAF subgroup marker genes and patient prognosis. We found that both C2 and C4 subgroups were negatively correlated with patient survival (**Figures 6C–E**).

### CAF subclusters exhibited distinct immunotherapy

**Figure 7:**
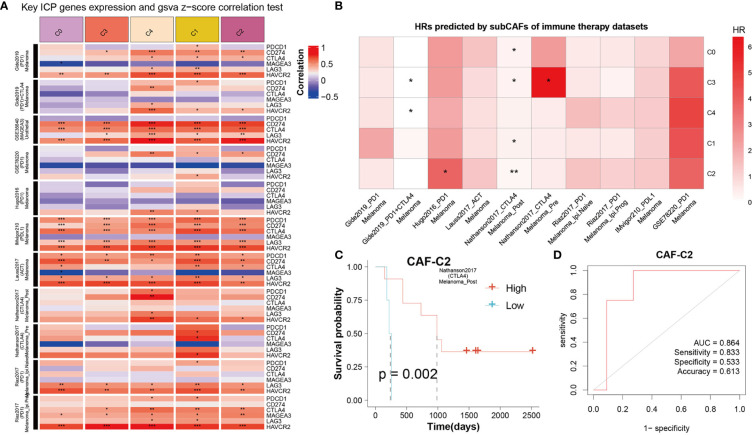
The correlation between CAF clusters and immune therapy. **(A)** Key ICP genes expression and GSVA z-score correlation test. Red means positive correlation, and blue means negative correlation. *P < 0.05, **P < 0.01, and ***P < 0.001. **(B)** HRs predicted by sub-CAFs of immune therapy datasets. The color represents the HR value. *P < 0.05 and **P < 0.01. **(C)** K-M curve for Nathanson2017_CTLA4 immunotherapy dataset based on CAF-C2 scoring. **(D)** The diagnostic model for immunotherapy response based on CAF-C2.

To get the immune features of the subset of single-cells, 10 cohorts with different tumors before or after immunotherapy (ICB) were collected in TIDE database to further analysis. The results showed that each sub-CAF had different levels of immunotherapy response (**Figure 7**). The expression of some ICP gene HAVCR2 was positively correlated with the GSVA z-score of these CAF subclusters (**Figure 7A**). We calculated the cell subset score of each sample in the immune therapy dataset for five CAF clusters and analyzed the correlation with prognosis by univariate cox analysis (**Figure 7B**). From **Figure 7B**, Nathanson2017_CTLA4 was found to have prognostic value in the four CAF clusters (CAF-C0, CAF-C1, CAF-C2, and CAF-C3). Therefore, we selected the Nathanson2017_CTLA4 immunotherapy dataset for CAF-C2 scoring, divided into high and low groups, and drew the K-M curve, from which we observe the poor prognosis of the low group (**Figure 7C**). We also developed a diagnostic model for immunotherapy response, as shown in **Figure 7D**.

### Quantitative real-time PCR

**Figure 8:**
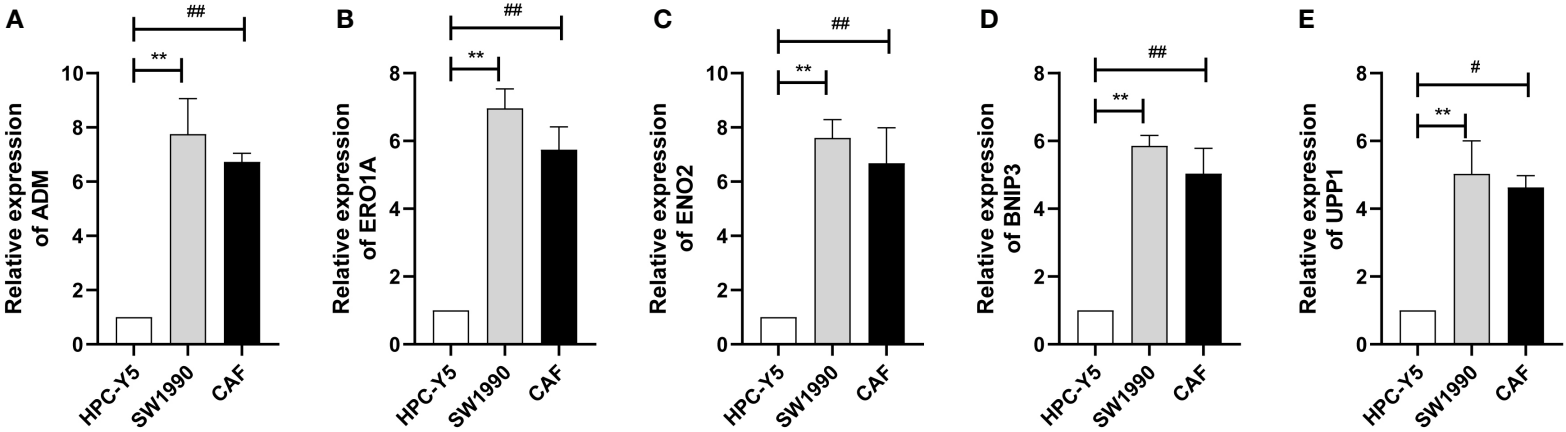
Quantitative real-time PCR. **(A–E)** Quantitative real-time PCR assays using cell lines for ADM **(A)**, ERO1A **(B)**, ENO2 **(C)**, BNIP3 **(D)**, and UPP1 **(E)**. **P < 0.01; ^#^P < 0.05; ^##^P < 0.01.

We selected the marker genes (ADM, ERO1A, ENO2, BNIP3, and UPP1) of CAF-C2 to detect their expression in human pancreatic CAF-stellate cell (CAF118), human pancreatic cell (HPC-Y5), and human pancreatic cancer cell line (SW1990). Compared with HPC-Y5, ADM (**Figure 8A**), ERO1A (**Figure 8B**), ENO2 (**Figure 8C**), BNIP3 (**Figure 8D**), and UPP1 (**Figure 8E**) were significantly higher expressed in SW1990 and CAF118.

## Discussion

PAAD is an aggressive malignancy, of which 95% are PDAC. In recent years, its morbidity and mortality rates have increased by an average of 0.3% per year due to changes in lifestyle and factors such as aging population and increased life expectancy (40). Because of the lack of specific symptoms and biological markers, early diagnosis of PAAD is very difficult. PAAD progresses rapidly and is inoperable by the time most patients are diagnosed (41). At the same time, pancreatic cancer is not sensitive to most treatments (42), so its prognosis is extremely poor (43). Some studies have shown that the unique interaction network and high heterogeneity of pancreatic cancer cells and that their microenvironment may play an important role in the origin, progression, and drug resistance of pancreatic cancer cells, and elucidating the inherent complex mechanisms has completed the common goal of scholars in this field (44, 45). Despite a lot of work, the results have been poor, with PAAD showing the lowest improvement in 5-year survival in recent years compared with other cancers (46). One of the important reasons lies in the limitations of traditional research methods in exploring the heterogeneity of tumors. Single-cell sequencing technology brings hope to break through this dilemma. It can deeply analyze the distribution, status, and interaction of different subgroups of cells, which makes up for some shortcomings of traditional sequencing technology and provides a new research method. At present, some studies based on single-cell sequencing technology have gradually achieved results and gradually gained a new understanding of the occurrence and progress of PAAD, providing possible targets for early diagnosis and effective treatment and promoting the development of precision medicine in the field of PAAD.

Molecular subtyping of PAAD is still in its early stage. In the clinical evaluation and prognostic analysis of PAAD, TNM stage and other clinical features are commonly used. However, because of individual differences in pancreatic cancer, there is no widely used molecular classification of pancreatic cancer that is associated with prognosis or has different sensitivity to treatment (47). Therefore, it is necessary to develop better methods for clinical diagnosis and prognosis assessment of PAAD so that patients can early detect cancer and take reasonable and effective treatment measures.

In recent years, with the continuous optimization and progress of the second-generation sequencing technology, the study of tumor bioinformatics has developed rapidly (48). However, there are a lot of mesenchymal components in PAAD tissue, which often leads to direct sequencing or inaccurate sequencing results (49, 50). Genomics studies have revealed common genomic pathway changes in PAAD, as well as more common or targeted somatic mutations in addition to the four major driver genes (51–53). Waddell et al. found that unstable patients may be more suitable for treatment with drugs involved in genomic damage repair pathways, such as Poly ADP-ribose polymerase (PARP) inhibitors or platinum, compared with other three types (53). More studies on PAAD typing have focused on transcriptomics. Because of the high content and complex composition of mesenchyma in PAAD tissues, there are some differences in sequencing analysis results. Sequencing analysis of samples with high or enriched tumor cells showed that PAAD tended to be divided into two types: classical and basal-like (54, 55). Studies of samples with relatively low levels of tumor cells, however, showed that pancreatic cancer types tended to be more diverse (55–57). This may be mainly due to the complexity of interstitial components, such as the differences in immune cell infiltration and interstitial activation. Canonical and basal-like transcriptome types are of great significance in predicting the prognosis of patients, but they have not yet played a good role in the classification and guidance of specific clinical treatment.

To further illuminate the subtyping of PAAD based on CAFs, we used Seurat 1656 CAFs in GSE154778 and 6228 CAFs in CRA001160. Our data discriminated five CAF subclusters and corresponding marker genes. To explore the mechanism of these CAF subclusters involved in the development of PAAD, we assess the characteristics of signaling patterns for the five CAF subclusters and found that these CAF subclusters were all closely related to EMT and endocrine. RHIM et al. traced that PAAD cells could develop EMT and obtain mesenchymal phenotype through *in vivo* pedigree, some cells after EMT initiated stem cell program, and PAAD cells with CD24^+^CD44^+^ stem cell phenotype were more likely to enter the blood circulation and survive (58). Breast cancer cells can also exhibit fibroblast characteristics and have the ability to differentiate into myofibroblasts (59). Our results further suggested that CAFs may be derived from EMT. We found that the expression of metabolism-related genes of CAF in each subclusters was different. Metabolic changes are an important feature in the identification of cancer cells. Many studies have found that CAFs are associated with energy metabolism of cancer cells, and tumor cells can better adapt to their rapid growth by modifying the TME. Sun et al. found that hypoxia can improve the glycolysis activity of CAFs, and lactic acid in hypoxia CAFs, as a metabolic coupling between CAFs and breast cancer cells, can improve the mitochondrial activity of cancer cells through relevant signaling pathways, thus promoting the invasion of breast cancer cells (60). In addition, in autophagy-related paracrine mode, CAFs provide substrates (such as lactic acid, pyruvate, and ketone bodies) for adjacent cancer cells derived from their own excess glycolysis activity (61). Research has shown that, in breast cancer, prostate cancer, head and neck carcinoma and lymphoma, and tumor, the catabolism of fibroblasts, the anabolic metabolism coupling between cancer cells, and the metabolic coupling drive fibroblasts of oxidative stress, glycolysis, autophagy, and aging; the decomposition in the metabolic production of fibroblasts for tumor growth provides a rich nutrition of microenvironment. The formation of mitochondrial fuel (lactic acid, ketone bodies, fatty acids, glutamine, and other amino acids) through a local matrix promotes tumor growth (62). CAFs can play an important role in the progression of cancer cells through a variety of metabolic pathways, which may provide new strategies for the treatment of PAAD.

In summary, we evaluated the heterogeneity of subclusters based on CAF for PAAD. The signaling patterns, immune status, metabolism features, and enrichment pathways of these subclusters were estimated and determined. Nonetheless, some limitations of the current study should not be ignored. The number of cells from the databases obtained in this study is limited, which varies from patient to patient. Therefore, more sample size is needed to support the conclusion. In addition, further high-throughput single-cell sequencing analysis and *in vivo* studies should be used to confirm the conclusions of this study.

## Conclusions

We explored the heterogeneity of five subclusters based on CAF in signaling patterns, immune status, metabolism features, enrichment pathways, and prognosis for PAAD.

## Data availability statement

The original contributions presented in the study are included in the article/**Supplementary Material**. Further inquiries can be directed to the corresponding author.

## Author contributions

HY conceived, designed, and supervised the study. GZ performed data analysis. CW, JJ and WZ arranged the figures and drafted the manuscript. All authors contributed to the article and approved the submitted version.

## Acknowledgments

We are grateful to the data from The Cancer Genome Atlas.

## Conflict of interest

The authors declare that the research was conducted in the absence of any commercial or financial relationships that could be construed as a potential conflict of interest.

## Publisher’s note

All claims expressed in this article are solely those of the authors and do not necessarily represent those of their affiliated organizations, or those of the publisher, the editors and the reviewers. Any product that may be evaluated in this article, or claim that may be made by its manufacturer, is not guaranteed or endorsed by the publisher.

## Abbreviations

PAAD, pancreatic adenocarcinoma; CAF, cancer-associated fibroblast; PDAC, pancreatic ductal adenocarcinoma; TME, tumor microenvironment; ECM, extracellular matrix; GEO, Gene Expression Omnibus; TISCH, Tumor Immune Single-cell Hub; TCGA, The Cancer Genome Atlas; ICB, immune checkpoint blockade; CCA, canonical correlation analysis; DEG, differentially expressed gene; KEGG, Kyoto Encyclopedia of Genes and Genomes; GO, Gene Ontology; GSVA, gene set variation analysis; K-M, Kaplan–Meier; OS, overall survival; RFS, relapse-free survival; ROC, receiver operating characteristic.
